# Inequalities in Frailty Among Older Turkish and Moroccan Immigrants and Native Dutch: Data from the Longitudinal Aging Study Amsterdam

**DOI:** 10.1007/s10903-021-01169-9

**Published:** 2021-02-26

**Authors:** Emiel O. Hoogendijk, Maaike E. Muntinga, Sascha de Breij, Martijn Huisman, Silvia S. Klokgieters

**Affiliations:** 1 Department of Epidemiology & Data Science, Amsterdam Public Health Research Institute, Amsterdam UMC–location VU University medical center, P.O. Box 7057, Amsterdam, 1007MB the Netherlands; 2 Department of Ethics, Law and Humanities, Amsterdam UMC–location VU University medical center, Amsterdam, the Netherlands; 3 Faculty of Social Sciences, Department of Sociology, Vrije Universiteit Amsterdam, Amsterdam, the Netherlands

**Keywords:** Frail older adults, Migration, Health inequalities, Frailty index

## Abstract

Very few studies have investigated frailty among older immigrants in Europe. The aim of the current study was to investigate inequalities in frailty in young-olds related to gender, educational level and country of origin, as well as intersections between these characteristics. Cross-sectional data were used from older Turkish and Moroccan immigrants (n = 466) and native Dutch (n = 1,020), all aged 55–65 years and participating in the Longitudinal Aging Study Amsterdam. Frailty was assessed with a 30-item frailty index, based on the deficit accumulation approach. Frailty was higher among women, lower educated, and people with a migration background. Of all groups considered, frailty levels were the highest among Turkish immigrants. No statistically significant interaction effects between gender, educational level and country of origin were found. When targeting frailty interventions, special attention should be devoted to older immigrants, as they are the most vulnerable group with the highest frailty levels.

## Background

As the global population is aging, the attention for the concept of frailty is increasing [1]. Frailty is an age-related condition, that is usually defined as a decline in reserve capacity in multiple physiological systems and an increased vulnerability to stressors [2]. Frailty is of relevance for both clinical practice and public health, as it is associated with adverse health outcomes, such as mortality, and increased healthcare costs [1].

Within the older population, variability in frailty exists. Differences in frailty by gender and socioeconomic position have been widely observed, with higher frailty levels among women and those with a lower socioeconomic position (e.g. lower education) [3–5]. Inequalities in frailty may also be determined by migration background and minority position. So far, most studies on racial and ethnic inequalities in frailty have been conducted in the US. These studies showed that older adults with a migration background and/or minority position (e.g. Hispanics, African Americans) generally have higher frailty levels compared with European Americans [6–8].

In Europe, frailty among older adults with a migration background received much less attention. After the Second World War, there were large groups of young individuals from the Mediterranean, including Turkey and Morocco, who migrated to Western and Northern Europe [9]. Reasons for migration vary, but many non-Western immigrants settled because of occupational opportunities. This first generation of migrant workers is now reaching older age, and is therefore of interest for health policy makers [10]. The few studies that have reported on frailty in older immigrants in Europe were based on the Survey of Health, Ageing and Retirement in Europe (SHARE) in various countries and the TOPICS-MDS dataset in the Netherlands [11–13]. However, in both studies, older immigrants were not purposely sampled, which may have led to selective samples. People with migrant background are structurally underrepresented in population-based studies in Europe, because of excluding sampling criteria such as speaking the native language [11, 12]. Moreover, all previous studies focused on one specific determinant of inequalities in frailty (i.e. gender, socioeconomic position, ethnic background), and did not look at intersections between these determinants. This is important because it is known that aspects of sociocultural identity are not experienced in isolation but rather that they are experienced simultaneously [14]. By looking at determinants of inequalities in frailty using intersectional methodology, specific vulnerable groups could be identified that need more attention in health policy [15–17].

The Longitudinal Aging Study Amsterdam (LASA) is an ongoing cohort study among older adults in the Netherlands [18, 19]. In 2013–2014, a cohort of older adults born in Turkey and Morocco was added to the LASA study. This provides unique opportunities to compare frailty among older adults with a migration background with native Dutch older people. Therefore, using data from older Turkish and Moroccan immigrants and native Dutch in LASA, the aim of the current study was to investigate inequalities in frailty related to gender, educational level and country of origin, as well as intersections between these characteristics.

## Methods

### Participants

We used data from LASA, an ongoing cohort study on physical, emotional, cognitive and social functioning of older adults in the Netherlands. Details on the LASA sampling and measurements have been published previously [18, 19]. In short, the study started in 1992 with a survey among older adults aged 55–84 years, based on a representative sample of the Dutch older population. Participants are interviewed in their homes approximately every 3 years. The data collection consists, amongst others, of a computer-assisted face-to-face interview and clinical tests. Two refresher cohorts aged 55–64 years were added to the study in 2002–2003 and 2012–2013, exactly 10 and 20 years after the start of LASA. Furthermore, a cohort of older adults born in Turkey and Morocco (first generation immigrants with a Dutch citizenship) was added to the study in 2013–2014 [19]. This was done to get better insight into aging and functioning of older non-Western immigrants living in the Netherlands. As of the 1960s, mainly male Turkish and Moroccan immigrants came to the Netherlands to perform (mostly) physical manual labor. Later, many wives and children from Turkey and Morocco followed these men to the Netherlands. Nowadays, they comprise the third and second largest groups of older non-Western immigrants living in the Netherlands [19]. These groups are often not represented in study samples among the general older population. Therefore, it was decided to purposely sample a cohort of older adults with a Turkish or Moroccan background, and to perform measurements that were largely comparable to those of the original LASA cohorts. The data collection for the immigrant cohort was conducted by trained interviewers of the same ethnic background in Dutch, Turkish, Moroccan Arabic (Darija) or Berber language (Tarifit). LASA is conducted in line with the Declaration of Helsinki and was approved by the medical ethics committee of the VU University medical center. All participants provided informed consent.

In the current study, we used cross-sectional data from the LASA sample of Turkish and Moroccan immigrants and native Dutch aged 55–65 years, collected between 2012 and 2014. The sample of older adults from Turkish and Moroccan origin consisted of 478 men and women born between 1948 and 1957. Data from the native Dutch come from the second refresher cohort that was added in 2012–2013. This cohort consisted of 1,023 men and women who were also born between 1948 and 1957. Due to some missing values on frailty (n = 10) and level of education (n = 5), the final analytical sample consisted of 1,486 people, of which 1,020 native Dutch and 466 Turkish and Moroccan immigrants.

### Measures

Frailty was measured by a 30-item frailty index based on the deficit accumulation approach. We followed the standard procedure described by Searle et al. [20] to create the frailty index. A frailty index counts signs, symptoms, diseases and disabilities that are related with age. The content is not fixed and may differ across studies, as long as specific requirements are met, such as a minimum of 30 deficits from multiple domains or organ systems [20, 21]. In LASA, a 32-item frailty index was created in 2017, and validated for predicting mortality [22]. This frailty index consisted of health deficits from physical, mental and cognitive domains. Due to some differences in questionnaires between the general LASA cohort and the sample of older immigrants [19], we slightly adapted the original LASA frailty index, and had to remove two items (two chronic diseases). This resulted in a 30-item frailty index (details are provided in Table 1). All deficits were scored between 0 and 1, with 0 indicating that a deficit is not present, and 1 indicating the presence of the deficit. For the calculation of the frailty index, a maximum of 20% missing variables is allowed (≤ 6 items), to enable maximum use of available data [23]. However, most people in the current study had no missing variables on the frailty index (96.6% of the sample). A frailty score was calculated by summing the health deficit scores and then divide them by the total number of items that were measured in a person (taking into account any missing items). For example, if a person presents with 6 health deficits out of 30 measured items, the frailty index score is 6/30 = 0.20. In addition to the continuous frailty index score, we also created a binary frailty indicator, by using the commonly used cut-point of ≥ 0.25 [24].

**Table 1:** Overview of the variables included in the 30-item frailty index

#	Deficit	Cut-off values
1	Cardiac disease	No = 0, Yes = 1
2	Peripheral arterial disease	No = 0, Yes = 1
3	Stroke	No = 0, Yes = 1
4	Diabetes	No = 0, Yes = 1
5	Lung disease	No = 0, Yes = 1
6	Cancer	No = 0, Yes = 1
7	Arthritis	No = 0, Yes = 1
8*	Hearing: follow conversation with or without hearing aid	Yes, without difficulty = 0, Yes, with some difficulty = 0.33, Yes, with much difficulty = 0.66, No = 1
9*	Vision: recognize face from 4 m with or without glasses	Yes, without difficulty = 0, Yes, with some difficulty = 0.33, Yes, with much difficulty = 0.66, No = 1
10	Walk up/down staircase 15 steps without resting	Yes = 0, Yes, with some difficulty = 0.25, Yes, with much difficulty = 0.50, Only with help = 0.75, No = 1
11	Dress/undress self	Yes = 0, Yes, with some difficulty = 0.25, Yes, with much difficulty = 0.50, Only with help = 0.75, No = 1
12	Sit down/stand up from chair	Yes = 0, Yes, with some difficulty = 0.25, Yes, with much difficulty = 0.50, Only with help = 0.75, No = 1
13	Cut own toenails	Yes = 0, Yes, with some difficulty = 0.25, Yes, with much difficulty = 0.50, Only with help = 0.75, No = 1
14	Walk outside 5 min without stopping	Yes = 0, Yes, with some difficulty = 0.25, Yes, with much difficulty = 0.50, Only with help = 0.75, No = 1
15	Use of transportation	Yes = 0, Yes, with some difficulty = 0.25, Yes, with much difficulty = 0.50, Only with help = 0.75, No = 1
16*	Take a bath/shower	Yes = 0, Yes, with some difficulty = 0.25, Yes, with much difficulty = 0.50, Only with help = 0.75, No = 1
17	How is your health in general?	Excellent = 0, Good = 0.25, Fair = 0.50, Sometimes good/bad = 0.75, Poor = 1
18	Feel depressed (CES-D)	Rarely or never = 0, Some of the time = 0.33, Occasionally = 0.66, Mostly or always = 1
19	Feel everything is an effort (CES-D)	Rarely or never = 0, Some of the time = 0.33, Occasionally = 0.66, Mostly or always = 1
20	Feel happy (CES-D)	Mostly or always = 0, Occasionally = 0.33, Some of the time = 0.66, Rarely or never = 1
21	Feel lonely (CES-D)	Rarely or never = 0, Some of the time = 0.33, Occasionally = 0.66, Mostly or always = 1
22	Enjoy life (CES-D)	Mostly or always = 0, Occasionally = 0.33, Some of the time = 0.66, Rarely or never = 1
23	Could not get going (CES-D)	Rarely or never = 0, Some of the time = 0.33, Occasionally = 0.66, Mostly or always = 1
24	Physical activity (LAPAQ)	High, 4 activities = 0, 3 activities = 0.25, 2 activities = 0.50, 1 activity = 0.75, No activity = 1
25	Memory complaints	No = 0, Yes = 1
26	Orientation time (MMSE)	Five correct = 0, One wrong = 0.50, Two or more wrong = 1
27	Orientation place (MMSE)	Five correct = 0, One wrong = 0.50, Two or more wrong = 1
28	Recall (MMSE)	Three correct = 0, Two correct = 0.50, One or zero correct = 1
29*	Drawing test (MMSE)	Correct = 0, Wrong = 1
30	Gait speed (6 m)	Normal = 0, Slow (> 10 s) or physically unable = 1

*Item adapted compared to original LASA frailty index [22]

Other variables included in the analyses were age (in years), gender, educational level, and country of origin (native Dutch vs. migrant background (i.e. born in Turkey or Morocco)). Educational level was a categorical variable with three groups: low (elementary school or less), medium (lower vocational or general intermediate education) and high (intermediate vocational education, general secondary school, higher vocational education, college or university).

### Analysis

First, descriptive analyses were performed to show the characteristics of the study sample, for the total sample and by country of origin (migrant background yes/no). This also includes the main characteristics of the frailty index (mean, median, prevalence). To gain insight into the distributions of the frailty index, we provided histograms. Next, we investigated differences in frailty related to gender, educational level and country of origin, and interactions between these characteristics. We did this for both frailty levels (continuous frailty index score) and frailty prevalence (binary frailty indicator). Linear regression analyses were performed with a log-transformed (natural log) frailty index score as outcome, since the distribution of the continuous frailty index score of the total study sample was slightly skewed to the right. Logistic regression analysis was used for the binary frailty indicator. For both outcomes, four models were tested: a model with age, gender, educational level and country of origin (Model 1), and models that additionally included an interaction term between gender and education (Model 2), an interaction term between gender and country of origin (Model 3), or an interaction term between education and country of origin (Model 4). The interaction effects provide insight into intersections between sociodemographic variables and aspects of sociocultural identity in relation to frailty. In sensitivity analyses, we repeated the main analyses (Model 1) with an adapted variable for country of origin, in which we considered Turkish and Moroccan immigrants as separate groups, because of the differences in sociocultural context between these groups. All analyses were performed in SPSS version 26 (IBM corp, Armonk, NY, USA).

## Results

The characteristics of the study sample are shown in Table 2. Overall, the mean age of the sample was 60.5 years, with a mean age of 60.9 years among older adults with a migrant background, and a mean age of 60.3 years in native Dutch. Overall, 48.5% of the study sample were women (41.6% among older adults with a migrant background and 51.6% among native Dutch). The majority of the immigrants was lower educated (72.3%), while the majority of the native Dutch was higher educated (57.2%). Of the immigrant sub-sample, 56% were from Turkey and 44% from Morocco.

**Table 2:** Descriptive statistics of the sample

	Total	Immigrants	Native Dutch
	n = 1,486	n = 466	n = 1,020
Age (years), mean (SD)	60.5 (3.0)	60.9 (3.0)	60.3 (2.9)
Gender (female), n (%)	720 (48.5)	194 (41.6)	526 (51.6)
Educational level			
Low, n (%)	441 (29.7)	337 (72.3)	104 (10.2)
Medium, n (%)	397 (26.7)	64 (13.7)	333 (32.6)
High, n (%)	648 (43.6)	65 (13.9)	583 (57.2)
Country of origin			
Turkey, n (%)	261 (17.6)	261 (56.0)	–
Morocco, n (%)	205 (13.8)	205 (44.0)	–
Native Dutch, n (%)	1,020 (68.6)	–	1,020 (100)
Frailty index score, range 0–1			
Mean (SD)	0.165 (0.126)	0.265 (0.143)	0.119 (0.085)
Median (IQR)	0.130 (0.075–0.223)	0.247 (0.151–0.361)	0.097 (0.061–0.155)
Frailty prevalence			
Frailty index ≥ 0.25, n (%)	318 (21.4)	231 (49.6)	87 (8.5)

**Fig. 1:**
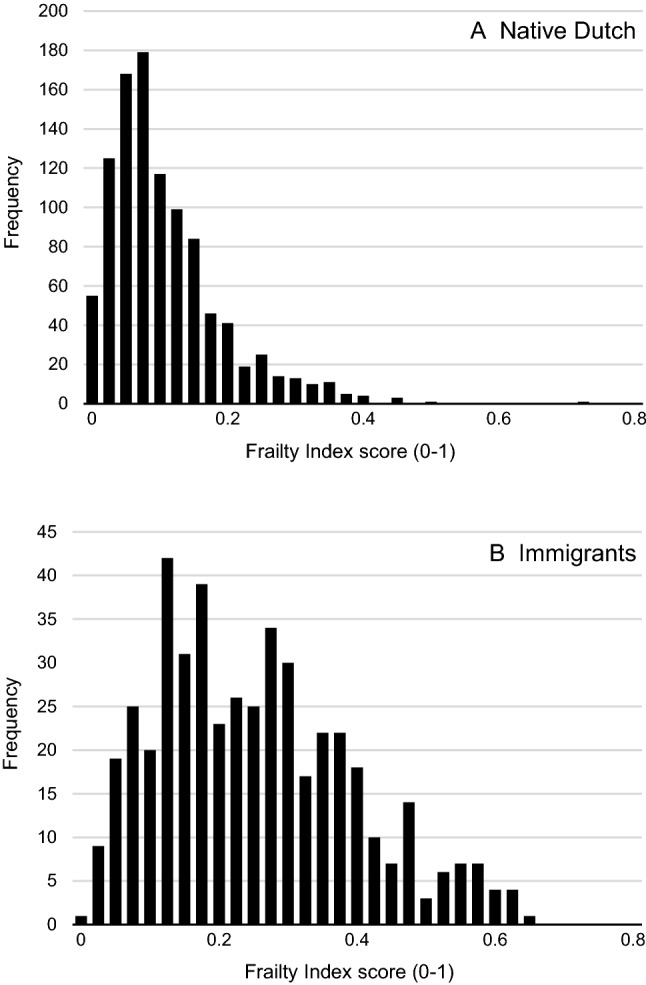
Distribution of the frailty index for (**a**) native Dutch and (**b**) Turkish and Moroccan immigrants

The distribution of the frailty index of native Dutch people is skewed to the right (Fig. 1, panel A). Among immigrants (panel B) the frailty index is more normally distributed. As shown in Table 2, the overall median frailty index score for the total sample was 0.130 (IQR = 0.075–0.223), ranging from 0.247 (IQR = 0.151–0.361) among immigrants to 0.097 (IQR = 0.061–0.155) among native Dutch. The frailty prevalence for the total sample was 21.4%, and this was higher among immigrants (49.6%) than among native Dutch (8.5%).

For the log-transformed continuous frailty index score, univariable linear regression analyses revealed that a higher age, being female, low education, medium education and having a migrant background were all associated with higher frailty levels (not shown in Table). In the multivariable linear regression analyses (Table 3, Model 1), the associations of gender (B = 0.170, SE = 0.033, p < 0.001), low education (B = 0.415, SE = 0.050, p < 0.001), medium education (B = 0.099, SE = 0.041, p = 0.02) and country of origin (having a migrant background, B = 0.583, SE = 0.046, p < 0.001) with frailty levels remained statistically significant.

**Table 3:** Multivariable linear regression: inequalities in log-transformed frailty index scores

	Model 1		Model 2		Model3		Model 4	
	B (SE)	p	B (SE)	p	B (SE)	p	B (SE)	p
Age (years)	0.007 (0.006)	0.21	0.007 (0.006)	0.20	0.007 (0.006)	0.21	0.007 (0.006)	0.21
Gender (female)	0.170 (0.033)	< 0.001	0.216 (0.050)	< 0.001	0.159 (0.040)	< 0.001	0.170 (0.033)	< 0.001
Educational level^a^								
Low	0.415 (0.056)	< 0.001	0.537 (0.125)	< 0.001	0.413 (0.050)	< 0.001	0.415 (0.068)	< 0.001
Medium	0.099 (0.041)	0.02	0.217 (0.128)	0.09	0.099 (0.041)	0.02	0.100 (0.044)	0.02
Country of origin (migrant background)	0.583 (0.046)	< 0.001	0.584 (0.046)	< 0.001	0.533 (0.111)	< 0.001	0.588 (0.083)	< 0.001
Gender × low education^b^			− 0.083 (0.078)	0.29				
Gender × medium education^b^			− 0.079 (0.081)	0.33				
Gender × country of origin					0.036 (0.072)	0.62		
Low education × country of origin^c^							− 0.004 (0.110)	0.97
Medium education × country of origin^c^							− 0.011 (0.120)	0.93

B = regression coefficient, SE = Standard error

^a^High educational level is the reference group

^b^Gender × high education is the reference group; ^c^High education × country of origin is the reference group

When using the binary frailty indicator as outcome measure in univariable logistic regression analyses, frailty prevalence was generally higher in those with a higher age, among women, lower educated, medium educated and among people with a migrant background. In multivariable logistic regression analyses (Table 4, Model 1), statistically significant associations remained for gender (OR = 1.82, 95% CI = 1.35–2.44), low education (OR = 2.81, 95% CI = 1.88–4.20) and country of origin (having a migrant background, OR = 6.59, 95% CI = 4.60–9.44).

**Table 4:** Multivariable logistic regression: inequalities in frailty prevalence (frailty index ≥ 0.25)

	Model 1		Model 2		Model3		Model 4	
	OR (95% CI)	p	OR (95% CI)	p	OR (95% CI)	p	OR (95% CI)	p
Age (years)	1.02 (0.98–1.08)	0.35	1.02 (0.98–1.08)	0.35	1.02 (0.98–1.08)	0.35	1.02 (0.97–1.07)	0.36
Gender (female)	1.82 (1.35–2.44)	< 0.001	1.45 (0.82–2.55)	0.20	1.88 (1.18–2.99)	< 0.01	1.82 (1.35–2.45)	< 0.001
Educational level^a^								
Low	2.81 (1.88–4.20)	< 0.001	1.75 (0.59–5.17)	0.31	2.82 (1.88–4.21)	< 0.001	3.04 (1.65–5.60)	< 0.001
Medium	1.31 (0.87–1.98)	0.20	0.88 (0.23–3.32)	0.84	1.31 (0.86–1.98)	0.20	1.53 (0.93–2.51)	0.09
Country of origin (migrant background)	6.59 (4.60–9.44)	< 0.001	6.50 (4.54–9.32)	< 0.001	7.22 (2.69–19.35)	< 0.001	8.51 (4.53–15.99)	< 0.001
Gender × low education^b^			1.38 (0.69–2.75)	0.36				
Gender × medium education^b^			1.31 (0.57–2.99)	0.53				
Gender × country of origin					0.94 (0.52–1.72)	0.85		
Low education × country of origin^c^							0.77 (0.33–1.77)	0.53
Medium education × country of origin^c^							0.60 (0.24–1.47)	0.26

OR = Odds ratio, 95% CI = 95% Confidence interval

^a^High educational level is the reference group

^b^Gender × high education is the reference group; ^c^High education × country of origin is the reference group

Finally, interaction effects were studied to gain insight into intersections between the various characteristics. No statistically significant interaction effects were found in the analyses of both the log-transformed frailty index score (Table 3, Models 2,3,4) and the binary frailty measure (Table 4, Models 2,3,4).

**Fig. 2:**
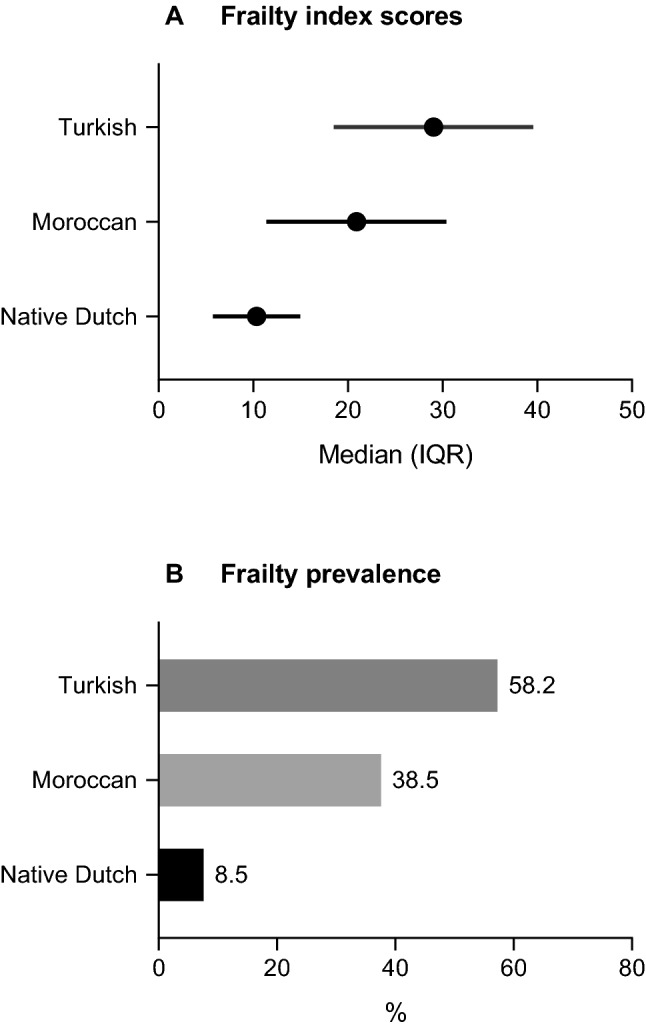
Sensitivity analyses: differences between native Dutch, Moroccan and Turkish adults aged 55–65 years in (**a**) frailty index scores and (**b**) frailty prevalence (frailty index ≥ 0.25). Continuous frailty index scores (panel A) were multiplied by 100 for interpretation purposes

In sensitivity analyses, we repeated the main analyses (Model 1) in which we considered Turkish and Moroccan immigrants separately (not shown in Table). These analyses revealed that, compared with native Dutch, Turkish immigrants had the highest frailty scores (B = 0.714, SE = 0.053, p < 0.001) or frailty prevalence (OR = 9.33, 95% CI = 6.25–13.92) followed by Moroccan immigrants, who also had higher frailty scores (B = 0.431, SE = 0.056, p < 0.001) or frailty prevalence (OR = 4.33, 95% CI = 2.84–6.60) compared to native Dutch. Figure 2 illustrates the differences between the three groups in continuous frailty scores (panel A) and frailty prevalence (panel B).

## Discussion

In this study, we investigated frailty among Turkish and Moroccan immigrants and native Dutch aged 55–65 years in the Netherlands. We observed higher frailty scores among women, those with lower educational levels, and among people with a migration background. This pertained to both frailty levels and frailty prevalence (frailty index score ≥ 0.25). Of all groups considered, frailty scores were highest among Turkish immigrants. Studying intersections between various socio-demographic characteristics did not reveal additional high-risk groups.

Our findings corroborate and extend previous studies. We showed that gender, educational level and migration background are all important determinants of frailty in young-old adults. Their associations with frailty remained in multivariable models that included all these characteristics and age as covariates. Previous studies in Europe did not investigate these determinants of frailty simultaneously. Higher frailty levels among women and lower educated people have often been observed [3, 4, 25–28]. Frailty among older immigrants in Europe received much less attention, but our findings point in the same direction as previous studies: older immigrants are more frail than older adults from native populations [11–13]. When comparing the frailty levels of the immigrants in the current sample (aged 55–65 years) with previously published reference values, it can be seen that their frailty corresponds to frailty levels of people aged 82 years and over in the Dutch general older population [22].

The current study revealed large disparities in frailty between native Dutch and immigrants of Turkish and Moroccan origin. There are various mechanisms that may explain poorer health of older adults with a migration background, such as lack of access to culturally safe and competent care, stigma and discrimination in the health care system, and socioeconomic inequalities. These may also explain the higher frailty levels among immigrants in later life, as observed in the current study. First, immigrants may have limited healthcare access, as a result of language barriers, or because of the fact that healthcare is not adapted to the specific needs of immigrants [29]. This may increase multi-system health decline in older immigrants, as captured by the frailty concept. Second, immigrants often experience poverty and structural challenges such as discrimination and prejudice [30, 31]. These are all factors that may have an adverse effect on physical and mental health. Third, it has been suggested that life course factors play an important role in the development of frailty in later life. Amongst others, adverse childhood exposures such as poverty may be related to frailty in midlife and beyond [32, 33]. When a person moves to another country, these risk factors from earlier stages in the life course may still affect frailty levels in later life.

Frailty is a condition with serious consequences in terms of adverse outcomes, including falls, fractures, loneliness, reduced quality of life, hospitalization, and mortality [1, 2]. Therefore, reducing or preventing frailty is of major importance [34]. The findings of the current study may have practical implications. The results suggest that, when developing interventional strategies to reduce frailty among older adults, special attention should be given to vulnerable groups, such as lower educated people and people with a migration background, in particular Turkish immigrants. However, to be able to develop specific interventions for targeting frailty among older immigrants, more research is needed on the mechanisms leading to the high frailty scores among Turkish and Moroccan immigrants. Insight into these mechanisms is essential to formulate targeted and ecologically valid policy recommendations.

This study has several strengths. We investigated frailty among older immigrants in Europe, a topic that has rarely been studied before in this population, making use of a well-validated frailty instrument. We used data from a large cohort study among young-old adults in the Netherlands, which included a group of Turkish and Moroccan immigrants, that was purposely sampled. In other studies, older non-Western immigrants were included by chance, which resulted in selective samples that were relatively small (4% to 6% of the total study population) [11–13]. Furthermore, we investigated intersections of inequalities, where previous studies merely focused on a single determinant of inequalities [11–13, 25, 35].

Nevertheless, the current study also has a few limitations. First, our study was focused on inequalities in frailty, but—except for age—we did not include other potential confounders or factors that may explain these inequalities. This may be addressed in future research. Second, we analyzed educational level as socioeconomic determinant of inequalities in frailty, but we were not able to include other relevant indicators of socioeconomic position, such as income and occupational status. Education is an important determinant of frailty, as was confirmed by our results, and it is causally related to income level and occupational prestige. Nevertheless, it is well-known that pathways linking each socioeconomic indicator to health outcomes are partly unique. Third, although we had a sufficiently large study sample (n = 1,486), some sub-groups (e.g., higher educated immigrant women) were relatively small. This may have limited the performance of three-way interaction effects in the regression analyses. Fourth, since we only included Turkish and Moroccan immigrants living in the Netherlands, our findings may not be generalizable to other immigrant populations in the Netherlands, or older immigrants in other European countries. Finally, the current study is cross-sectional. Longitudinal data would be needed to see how frailty develops among older immigrants compared to native Dutch, and to investigate the extent to which the frailty index predicts adverse outcomes in these groups. However, longitudinal data has not yet been collected among the LASA immigrant cohort. Data on mortality will become available in the future, as vital status of all respondents may be retrieved from municipality registers [19].

## New Contribution to the Literature

This study among Turkish and Moroccan immigrants and native Dutch aged 55–65 years revealed inequalities in frailty related to gender, education and country of origin. The highest frailty levels were observed among older immigrants, and in particular among Turkish immigrants. Since frailty is a condition with serious consequences in terms of quality of life and adverse outcomes [1], targeted interventions to reduce or prevent frailty among older immigrants are needed [34]. However, additional research is required to understand the mechanisms underlying the higher frailty scores among immigrants.
